# Development and Usability Testing of a Mobile App–Based Clinical Decision Support System for Delirium: Randomized Crossover Trial

**DOI:** 10.2196/51264

**Published:** 2024-01-24

**Authors:** Jiamin Wang, Meihua Ji, Yuan Han, Ying Wu

**Affiliations:** 1 School of Nursing, Beijing University of Chinese Medicine, Beijing, China; 2 School of Nursing, Capital Medical University, Beijing, China; 3 Peking University First Hospital, Beijing, China

**Keywords:** delirium, 3D-CAM, older adults, clinical decision support system, nurse, 3-Minute Diagnostic Interview for Confusion Assessment Method-Defined Delirium

## Abstract

**Background:**

The 3-Minute Diagnostic Interview for Confusion Assessment Method–Defined Delirium (3D-CAM) is an instrument specially developed for the assessment of delirium in general wards, with high reported sensitivity and specificity. However, the use of the 3D-CAM by bedside nurses in routine practice showed relatively poor usability, with multiple human errors during assessment.

**Objective:**

This study aimed to develop a mobile app–based delirium assessment tool based on the 3D-CAM and evaluate its usability among older patients by bedside nurses.

**Methods:**

The Delirium Assessment Tool With Decision Support Based on the 3D-CAM (3D-DST) was developed to address existing issues of the 3D-CAM and optimize the assessment process. Following a randomized crossover design, questionnaires were used to evaluate the usability of the 3D-DST among older adults by bedside nurses. Meanwhile, the performances of both the 3D-DST and the 3D-CAM paper version, including the assessment completion rate, time required for completing the assessment, and the number of human errors made by nurses during assessment, were recorded, and their differences were compared.

**Results:**

The 3D-DST included 3 assessment modules, 9 evaluation interfaces, and 16 results interfaces, with built-in reminders to guide nurses in completing the delirium assessment. In the usability testing, a total of 432 delirium assessments (216 pairs) on 148 older adults were performed by 72 bedside nurses with the 3D-CAM paper version and the 3D-DST. Compared to the 3D-CAM paper version, the mean usability score was significantly higher when using the 3D-DST (4.35 vs 3.40; *P*<.001). The median scores of the 6 domains of the satisfactory evaluation questionnaire for nurses using the 3D-CAM paper version and the 3D-DST were above 2.83 and 4.33 points, respectively (*P*<.001). The average time for completing the assessment reduced by 2.1 minutes (4.4 vs 2.3 min; *P*<.001) when the 3D-DST was used.

**Conclusions:**

This study demonstrated that the 3D-DST significantly improved the efficiency of delirium assessment and was considered highly acceptable by bedside nurses.

## Introduction

Delirium is a common complication that is highly prevalent among hospitalized older adults; it can affect up to 40% of hospitalized older patients [1,2]. Delirium is associated with many adverse outcomes, including cognitive and functional impairment, increased hospital stay, care burden, and mortality, and therefore leads to increased care needs and poor prognosis [3,4]. The severity of the adverse consequences of delirium are positively correlated with the severity and duration of delirium [5]. Therefore, early recognition of delirium is essential for timely management of delirium to improve the patient’s prognosis [6].

Although many clinical guidelines and representative societies have recommended the use of standardized assessment tools for daily delirium screening in clinical practice, delirium is still poorly recognized among hospitalized older adults [6,7]. A previous study has shown that up to 66% of older adults with delirium went unrecognized in routine practice [8]. The possible reasons for underdiagnoses of delirium include communication barrier, inadequate use of the screening tools, and a lack of education on and conceptual understanding of delirium [9].

The Confusion Assessment Method (CAM) has been used as the reference standard for delirium screening [10]. However, the use of the CAM to assess delirium requires a combination of cognitive testing and subjective judgment based on clinical experience. Lemiengre et al [11] found that the sensitivity on the daily use of the CAM by bedside nurses was only 30%. Therefore, to overcome the feasibility issues associated with the use of the CAM, Marcantonio et al [12] developed the 3-Minute Diagnostic Interview for CAM-Defined Delirium (3D-CAM) based on the key features of the CAM and simplified the evaluation process. The 3D-CAM was perceived as easy to use, and it had a sensitivity of 92% to 100% and a specificity of 88% to 94% among studies that were tested in different ethnic groups [12-15]. Kuczmarska et al [15] has identified that the 3D-CAM is appropriate for delirium assessment in general wards.

However, a previous study has shown that the use of the 3D-CAM by bedside nurses in routine practice demonstrated relatively poor usability, with multiple errors (such as human error, misunderstanding of item content, and incomplete or missing nursing records) during assessment [16]. The assessment of alteration on attention and thinking must be combined with cognitive assessment. During the process, nurses need to memorize, calculate, and make a judgment according to the patient’s response, which is prone to human errors. Meanwhile, the patient’s consciousness status needs to be compared with the assessment results at admission by reviewing the patient’s medical records. With these identified challenges, nurses often fail to complete the assessment successfully due to communication problems and difficulties in finding or loss of related materials. Moreover, due to the limited knowledge level on delirium among clinical nurses, adding prompts or cues to the items can effectively improve nurses’ identification of delirium, in addition to providing training on basic delirium knowledge before the use of the 3D-CAM [16]. Therefore, there is an urgent need to develop and adopt innovative ways to promote prompt delirium assessment in routine practice both effectively and accurately.

Several studies have reported that a clinical decision support system (CDSS) with some degree of autonomy may help solve this issue and improve the accuracy and adequacy of delirium assessment among bedside nurses [17,18]. Marcantonio et al [19] developed a brief app-based delirium identification tool, and it has shown good performance. Based on the Confusion Assessment Method for Intensive Care Unit (CAM-ICU), mobile apps have also been developed for delirium screening of patients in intensive care units (ICUs), and they showed acceptable usability and accuracy when used by bedside nurses [20-22]. Therefore, in this study, we aimed to develop the Delirium Assessment Tool With Decision Support Function Based on the 3D-CAM (3D-DST) and to evaluate its usability among older patients by bedside nurses.

## Methods

### Ethical Considerations

The research protocol and secondary data analysis were approved by the institutional review committee of Capital Medical University (2015SY49). This was a substudy under a clinical trial, which was registered at the Chinese Clinical Registry (ChiCTR-IOR-17,010,368). Verbal informed consent was obtained from each participant before the start of the program, and participants had the right to withdraw during the study. The data were anonymized. The study protocol was safe and reliable and did not provide any compensation to the participants.

### Design and Development of the 3D-DST

#### Previous Work

The 3D-CAM was translated into Chinese in a previous study and validated by nurse researchers; it showed acceptable sensitivity and specificity among hospitalized Chinese older patients [23].

#### Phase 1: Analysis of Problems in the Use of the 3D-CAM Paper Version

The task walkthrough method was used to fully address the end users’ needs and achieve the overall goal of automatic delirium evaluation [16].

#### Phase 2: Design of the 3D-DST

In this phase, we first formed a multidisciplinary team that included experts with rich experience in delirium assessment. Bedside nurses, nursing researchers, software engineers, and user interface designers who were specialized in developing nursing information systems (NISs) were involved in the designing phase. The 3D-DST was designed and developed following the American Medical Informatics Association usability design principle [24]. Details of the design principle are shown in Multimedia Appendix 1.

### Evaluation Process Analysis and Optimization of the 3D-DST

Based on the evaluation content and rules of the original 3D-CAM, this study scrutinized and analyzed the 3D-CAM evaluation process, identified the best path, and reoptimized and standardized the 3D-CAM evaluation process. By optimizing the 3D-CAM evaluation process, only the necessary paths to support the delirium evaluation were reserved, unnecessary links were eliminated, and the assessment was standardized to reduce intermediate errors; , thus, a clinical decision-making system for delirium screening was established. Automatic evaluation logic jumps were incorporated into the system design according to the evaluation rules. Based on the problems identified by bedside nurses and the overall goals of the system, the key functional modules of the 3D-DST were initially drafted in mind-mapping software (Xmind software, version: 3.7.4.0; XMIND LTD).

### User Interface Design of the 3D-DST

#### Overview

The interface design was completed in several steps. First, we used AxureRP (Axure Rapid Prototyping) to draft the logical diagrams, workflow, functional components, and user interfaces of the 3D-DST. Second, we made a web page to facilitate communication with engineers and team members. This was achieved via WuliHub (a domestic data hosting and sharing platform), and the interface prototype diagram and interaction components drawn by AxureRP were uploaded into this web browser–based demonstration scheme. Via WuliHub, a set of HTML files were generated based on the interface prototypes and interaction schemes drawn by AxureRP; they were compressed and uploaded to the platform for easy sharing. Third, following the American Medical Informatics Association interface design principle, the order of the evaluation content of each interface was determined based on the evaluation content and the optimal evaluation process of the 3D-DST. Finally, the prototype design of the evaluation interface of the 3D-DST was completed with the identified functions, including evaluator registration, log-in authentication, and user log out. Corresponding functional assessment modules were also developed, along with the result-reporting interface. The 3D-DST was designed by following the existing delirium assessment system [23], with easy access and a friendly display; for example, the patient’s identification was obtained by scanning their wristband, and different color selection, buttons, and information composition were used to increase its feasibility and usability.

#### Phase 3: Architecture and Development of the 3D-DST

In this phase, the system architecture, databases related to personal information and assessment data, and the user interfaces were identified. The 3D-DST was developed to fit on Android-compatible devices (Huawei nova 3), as the personal digital assistant used in clinical practice was largely based on the Android system rather than the iOS system in China.

We used Java, Spring Boot, and RouYi-Vue to program the backend framework and Vue for the front-end framework. Mysql was used to formulate the databases. GitLab and Docker were used to release the 3D-DST. To make the 3D-DST system more stable, the model-view-viewmodel (MVVM) was used as the architecture scheme. The synchronization between view and model was completely automatic without human interference, the data maintenance was completely managed by the MVVM, and the operating environments were Linux and Windows [25,26]. To maintain information security, the 3D-DST set a cookie scheme with a time limit. The users needed to log in and verify their identity again when the cookie expired.

### Usability Evaluation of the 3D-DST

#### Overview

The usability of the 3D-DST was evaluated using the acceptance questionnaire, and the results were compared to the 3D-CAM paper version when it was used by bedside nurses. The questionnaire included 6 domains regarding usability: perceived usefulness, ease of use, ease of learning, trustworthiness, intention to use, and satisfaction. The performance of the app was evaluated on 4 domains and compared to that of the 3D-CAM paper version, which included the successful completion rate of delirium assessment (proportion of nurses who completed the assessment correctly when they used the 3D-CAM paper version and the 3D-DST), evaluation completion time (time used to complete the assessment), the number of mistakes made during assessment against the results from a researcher, and satisfactory evaluation using an acceptance questionnaire.

#### Design, Setting, and Participants

This study was conducted among 72 bedside nurses from 3 tertiary hospitals in Beijing, China. Eligible patients from 3 internal medical wards (neurology, respiratory, and cardiology) and 1 surgical ward (orthopedic) of the study hospitals were assessed by participating bedside nurses using both the 3D-DST and the original 3D-CAM paper version. All participating patients and bedside nurses provided informed consent before study initiation.

Following convenient sampling, bedside nurses who met the following criteria were included in the usability testing phase: (1) registered nurse with a valid license; (2) had more than 1 year of working experience and had been working continuously in their department for more than 3 months; and (3) willing to participate in this research. Hospitalized older adults who were aged 65 years or older and could communicate effectively in Mandarin were included in the study, and those with identified severe visual or hearing impairment were excluded from delirium assessment. Nurses who declined to participate during the study period were also excluded from the final analysis.

#### Usability Evaluation

The usability evaluation of both the 3D-CAM paper version and the 3D-DST was conducted using the usability testing questionnaire designed by Feng et al [27]. The content validity index scores for the 3 areas in the questionnaire (topic suitability, topic importance, and content clarity) were 1.00, 1.00 and 0.96, respectively. Since the dimensions for evaluating the usability of the CAM-ICU and 3D-CAM are similar, the questionnaire can also be used to evaluate the usability of the 3D-CAM. In this study, we revised several items of the questionnaire to make it suitable for the 3D-CAM paper version or the 3D-DST. For example, we changed the item “I think this tool meets the requirements of ICU nurses for a delirium assessment tool” to “I think this tool meets the requirements of nurses for a delirium assessment tool in general wards.” In addition, the term “CAM-ICU” in the items evaluating the ease of use was replaced with “3D-CAM.” The reliability of the revised questionnaire was tested for overall internal consistency, and the Cronbach α coefficient was 0.907. The questionnaire was rated on a 5-point Likert scale ranging from “1=strongly disagree” to “5=strongly agree.” A higher score indicated better user acceptance. The usability evaluation was evaluated on 6 domains: perceived usefulness, ease of use, ease of learning, trustworthiness, intention to use, and user satisfaction. To evaluate the acceptability of the 3D-DST, items to evaluate the interfaces of the app were added to the questionnaire, so the usability questionnaire contained 26 and 43 items for the 3D-CAM paper version and the 3D-DST, respectively.

**Figure 1.**
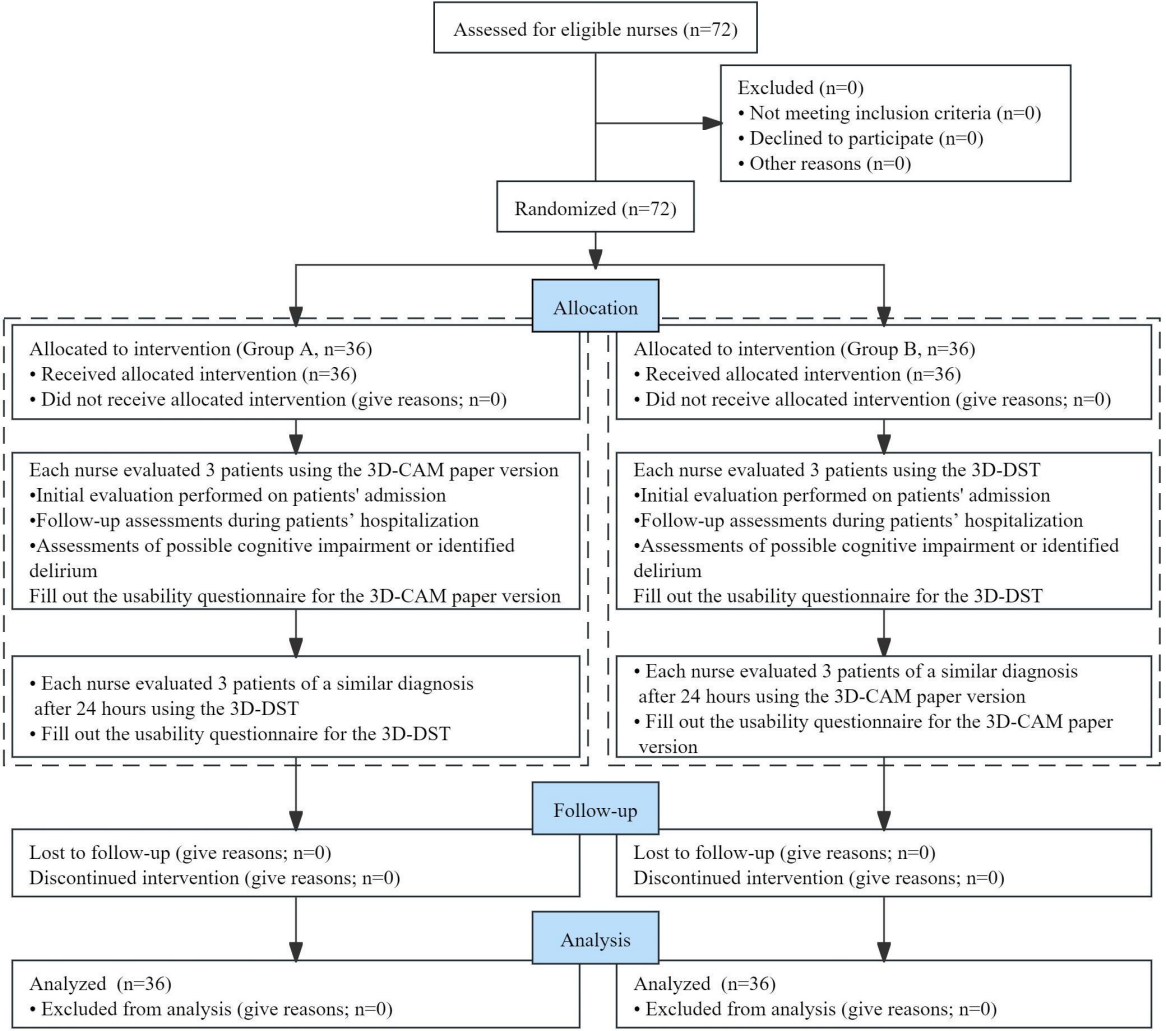
Flowchart of participant recruitment and participation. 3D-CAM: 3-Minute Diagnostic Interview for Confusion Assessment Method–Defined Delirium; 3D-DST: Delirium Assessment Tool With Decision Support Function Based on the 3-Minute Diagnostic Interview for Confusion Assessment Method–Defined Delirium.

Before study initiation, researchers used a computer program to generate random numbers and made an assignment sequential table. Eligible bedside nurses were numbered according to the order they participated in. One group of nurses (Group A) used the 3D-CAM paper version first to evaluate 3 patients (initial evaluation at admission and follow-up assessments during the patients’ hospitalization, including assessments of possible cognitive impairment or identified delirium). Subsequently, the same group of bedside nurses used the 3D-DST to evaluate patients with a similar admission diagnosis after 24 hours to avoid the impact of short-term memorization on the evaluation process. Conversely, nurses from the other group (Group B) used the 3D-DST first and then the 3D-CAM paper version second to evaluate 2 different sets of patients (3 patients per nurse). Nurses who participated in the study only assessed patients admitted in the wards where they worked. A nurse researcher conducted training sessions for all bedside nurses on the use of the 3D-CAM and the 3D-DST before their assessments; the nurse researcher also selected the eligible patients based on their admission diagnosis prior to being approached. Usability testing of both the 3D-CAM paper version and the 3D-DST was carried out when each set of assessment was completed (Figure 1).

#### Performance of the 3D-DST

During each assessment, 1 experienced nurse researcher observed the performance of bedside nurses and completed the patients’ delirium screening using the 3D-CAM paper version at the same time. The successful completion rate of delirium assessments in terms of the proportion of correctly identified delirium, whether using the 3D-CAM paper version or the 3D-DST by bedside nurses, was recorded. In addition, the number of mistakes made during the assessments (compared to the researcher’s assessment results) and the evaluation completion time were also recorded for each nurse. Nurses were blinded to the researcher’s assessment results.

#### Sample Size

The highest score for each item of the usability questionnaire was 5 points (the higher the score, the better the usability). We expected that an average score of 4 or more would be achieved when the 3D-DST was used by bedside nurses, which is 1 point higher than the average score of the 3D-CAM paper version. With the SD being 1.0 and α=.05, at least 54 bedside nurses were needed to achieve 90% power. Considering that a quarter of bedside nurses might not be able to complete the study, a final sample of 72 nurses was required for this study.

#### Data Analysis

SPSS software (version 21.0; IBM Corp) was used to perform the data analysis. Normally distributed variables were presented using the mean and SD, whereas nonnormally distributed variables were presented using the median and range. Categorical variables were presented with frequencies and proportions as appropriate. The *χ*^2^ test or Fisher exact test was used to compare the differences between nurses’ baseline data (different age groups, sex, etc) and the evaluation success rate. The comparison of evaluation completion time, the total usability score, and the impact of the sequential use of the 3D-CAM paper version and the 3D-DST between 2 groups were achieved using ANOVA. A nonparametric test was used to test the differences between the scores of each domain of the usability questionnaire and the order of using the 2 types of assessments. *P*<.05 was considered statistically significant.

## Results

### Design and Development of the 3D-DST

#### Phase 1: Analysis of Problems in the Use of the 3D-CAM Paper Version

Several problems were identified with the use of the 3D-CAM paper version by bedside nurses, including human errors, insufficient or incorrect understanding of the assessment contents, and incomplete or failed retrieval of the relevant information from the nursing record [16]. In developing the 3D-DST, the delirium evaluation process was simplified as unnecessary steps were automatically omitted after sorting out the procedural results related to specific features of the 3D-CAM. The evaluation processes of the 3D-CAM paper version and the 3D-DST are shown in Multimedia Appendices 2 and 3.

#### Phase 2: Design of the 3D-DST

**Figure 2.**
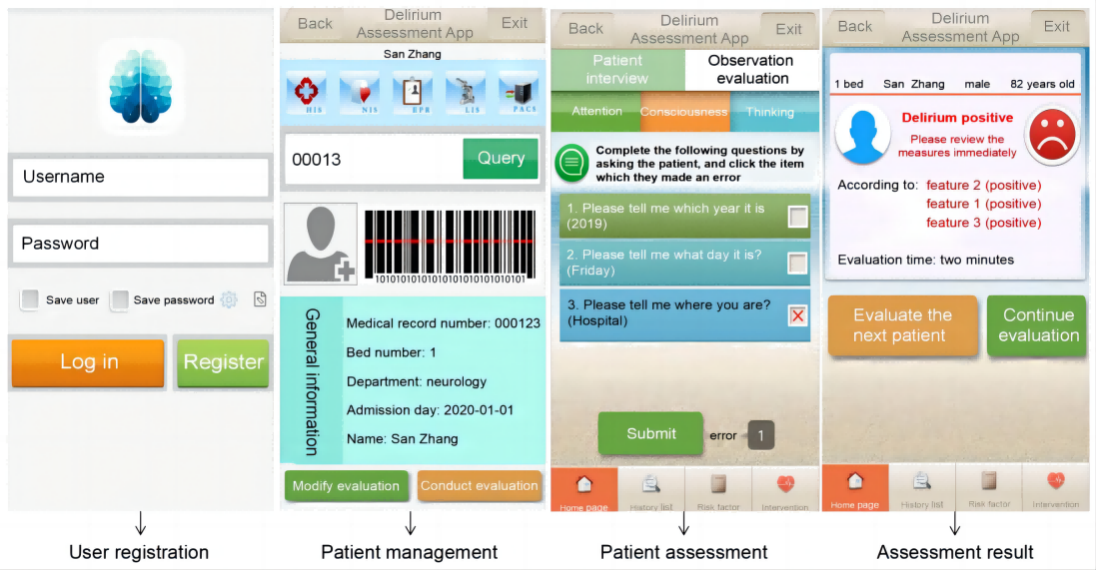
Interfaces of the Delirium Assessment Tool With Decision Support Function Based on the 3-Minute Diagnostic Interview for Confusion Assessment Method–Defined Delirium (3D-DST).

The 3D-DST was installed on mobile phones with an Android (8.1.0) system, 128 GB, 8-core processor, and 1.8 GHz. Three evaluation modules were incorporated into the 3D-DST, reflecting the inquiry, observation, and selective evaluations. The evaluation interfaces (Figure 2) of the 3D-DST included 8 evaluation pages and 16 different evaluation result interfaces (3 delirium-positive interfaces and 13 delirium-negative interfaces).

Auxiliary reminders based on the common mistakes reported by nurses in phase 1 were added to the system. In overcoming these burdens, the 3D-DST was developed to include reminders on the correct answers regarding items 1, 2, and 5. Nurses only needed to check whether the patient’s response was consistent with the reminder or not. As the 3D-DST can automatically record and retrieve previous evaluation results, item 22 was not displayed in the 3D-DST but was incorporated into the algorithm of the 3D-DST, and the result was generated automatically. To address issues associated with “incorrect understanding of item content,” cues were incorporated into the 3D-DST to facilitate better understanding of the contents of related items (11 through 20) during assessment.

In the development of the 3D-DST, we also included functions to automatically retrieve necessary information from the record for comparison, which was incorporated to the 3D-DST. Nurses using the 3D-DST do not need to manually search and compare the previous evaluation results, which could increase the successful completion rate of the assessment.

### Usability Evaluation of the 3D-DST

A total of 72 bedside nurses completed the usability testing of the 3D-DST. The demographic characteristics of participating bedside nurses are shown in Table 1. They were mainly female (n=67, 93%), with 44 (61%) nurses holding a bachelor’s degree. A total of 148 patients were evaluated by nurses in this study, with 98 (66%) being female and a mean age of 76 (SD 7.18) years.

**Table 1.** Demographic characteristics of the participating bedside nurses.

Characteristics		Group A (n=36), n (%)	Group B (n=36), n (%)	*P* value
**Age group (years)**				.93
	20-30	20 (56)	20 (56)	
	31-47	16 (44)	16 (44)	
**Sex**				>.99
	Female	33 (92)	34 (9)	
	Male	3 (8)	2 (56)	
**Education level**				>.99
	Junior college	12 (33)	13 (36)	
	College and above	24 (67)	23 (64)	
**Work experience (years)**				.71
	<5	15 (42)	16 (44)	
	5-10	11 (31)	9 (25)	
	≥11	10 (28)	11 (31)	

A total of 432 delirium assessments (216 pairs) were performed by the bedside nurses on 148 older adults with the 3D-CAM paper version and the 3D-DST, of which 38 (26%) older adults were confirmed as delirium positive by a nurse researcher using the 3D-CAM paper version.

The mean usability scores of bedside nurses using the 3D-CAM paper version and the 3D-DST were 3.40 (SD 0.43) and 4.35 (SD 0.31), respectively, with the score of the 3D-DST being 0.95 points higher than that of the 3D-CAM paper version (*P*<.001). The median scores of the 6 domains of the satisfactory evaluation questionnaire for nurses using the 3D-CAM paper version and the 3D-DST were above 2.83 points and 4.33 point, respectively. As shown in Table 2, the median scores on the ease of use, ease of learning, and trustworthiness were 1 point higher than the other 3 domains (*P*<.001). The total satisfactory score of the 3D-DST was higher than that of the 3D-CAM paper version (*P*<.001), whereas the order of assessments, whether using the 3D-CAM paper version first or the 3D-DST first, had no effect on the results.

**Table 2.** Comparison of the effectiveness and efficiency of the 3D-CAM^a^ paper version and the 3D-DST^b^.

Domain		Score, median (range)	*Z* value	*P* value
**Perceived usefulness**			–6.903	<.001
	3D-DST	4.57 (3.29-5.00)		
	3D-CAM paper version	3.86 (2.71-4.86)		
**Perceived ease of use**			–6.973	<.001
	3D-DST	4.33 (3.00-5.00)		
	3D-CAM paper version	2.83 (1.83-4.33)		
**Learnability**			–7.014	<.001
	3D-DST	4.33 (3.00-5.00)		
	3D-CAM paper version	3.33 (2.33-4.67)		
**Trustworthiness**			–6.697	<.001
	3D-DST	4.33 (3.00-5.00)		
	3D-CAM paper version	3.33 (2.00-5.00)		
**Intention to use**			–6.446	<.001
	3D-DST	4.40 (3.20-5.00)		
	3D-CAM paper version	3.67 (1.33-5.00)		
**Satisfaction**			–6.985	<.001
	3D-DST	4.33 (3.33-5.00)		
	3D-CAM paper version	3.60 (1.60-4.20)		

a 3D-CAM: 3-Minute Diagnostic Interview for Confusion Assessment Method–Defined Delirium.

b 3D-DST: Delirium Assessment Tool With Decision Support Function based on the 3-Minute Diagnostic Interview for Confusion Assessment Method–Defined Delirium.

The assessment success rate of the 3D-CAM paper version was a little lower compared to that of the 3D-DST (203/216, 94% vs 212/216, 98.1%; *P*=.045). The median time of assessment using the 3D-CAM paper version was 2.1 minutes longer than that of the 3D-DST (4.4 vs 2.3 min; *P*<.001). The overall performances of the 3D-DST and the 3D-CAM paper version are displayed in Table 3.

**Table 3.** Comparison of the performances between the 3D-CAM^a^ paper version and the 3D-DST^b^ by bedside nurses.

Performance		Nurses, n	Assessments, n	Group A	Group B	*P* value
**Successful completion rate, n/N (%)**						.045
	3D-CAM paper version	72	216	103/108 (95.4)	100/108 (92.6)	
	3D-DST	72	216	107/108 (99.1)	105/108 (97.2)	
**Human errors, n**						.62
	3D-CAM paper version	72	N/A^c^	6	9	
	3D-DST	72	N/A	2	3	
**Evaluation completion time (min), median (IQR)**						<.001
	3D-CAM paper version	72	203	4.45 (2.5-5.4)	4.35 (2.4-5.3)	
	3D-DST	72	212	2.25 (1.25-3.55)	2.35 (1.4-3.6)	

a 3D-CAM: 3-Minute Diagnostic Interview for Confusion Assessment Method–Defined Delirium.

b 3D-DST: Delirium Assessment Tool With Decision Support Function Based on the 3-Minute Diagnostic Interview for Confusion Assessment Method–Defined Delirium.

c N/A: not applicable.

## Discussion

### Principal Findings

This study described the development process of a CDSS based on the 3D-CAM and evaluated its usability in delirium screening among older patients. Our results demonstrated that the 3D-DST was perceived as highly satisfactory with acceptable usability when used by bedside nurses, and it improved the completion rate and reduced the evaluation completion time when bedside nurses used the app among older patients.

CDSSs are tools incorporated with a significant clinical knowledge base and are designed to provide users (health care professionals, patients, and caregivers) with an intelligent way to assist in clinical decision-making [28]. Previous studies have found that well-designed CDSSs are effective and can improve clinical outcomes and health processes [29]. Therefore, it is very important to ensure the quality of CDSSs and avoid unpleasant situations when deploying unreliable systems.

Functional suitability is a very important feature when developing CDSSs; it refers to the extent to which a system meets the stated and implied requirements through its functional components under certain conditions [30-32]. In our study, the 3D-DST is aimed to connect with the hospital information system or NIS, so that it can automatically retrieve the patients’ information via the hospital information system. The 3D-DST can obtain information by scanning the QR code on the patient’s wristband, which allows the 3D-DST to be easily integrated into the portable NIS and facilitates efficient delirium assessment by bedside nurses.

As indicated by the study results, the median scores on the ease of use, ease of learning, and trustworthiness of the 3D-DST were over 1 point higher than those of the 3D-CAM paper version. It was demonstrated that the development of the 3D-DST met the requirements of bedside nurses, improving the acceptability and usefulness of the screening tool. There were several possible reasons. First, the 3D-DST was designed through process optimization, which incorporated strategies such as automatic evaluation logic jumps, embedded prompts, automatic comparison function, etc. The design process made full use of information technologies, such as automatic recording, calculation, and other intelligent functions, that could effectively solve the problems identified by nurses when they used the 3D-CAM paper version. The 3D-DST was well accepted by bedside nurses, the burden on the memorization of information was reduced, and the ease of use of the 3D-CAM was improved. Second, nurses only needed to complete the evaluation process by following the interfaces and the embedded prompts. Nurses did not need to learn the specific instructions, and the system could automatically record, calculate, and output the results, therefore improving this tool’s learnability and scalability. Third, since the content of the 3D-CAM is mostly a routine assessment, nurses needed less training time to use the tool. Moreover, with reduced time to complete the delirium assessment, nurses perceived the 3D-DST as highly acceptable, which generated trustworthiness and solved the existing problems associated with the use of the 3D-CAM paper version.

The improvement of the perceived usefulness, intention to use, and satisfaction scores was less than 1 point when comparing the 3D-DST with the 3D-CAM paper version. This could be attributed to the following reasons. The scores on these 3 domains of the 3D-CAM paper version were considerably high. Perceived usefulness was mainly evaluated based on nurses’ knowledge of delirium assessment, whereas intention to use mainly referred to whether nurses were willing to use the tool and whether the nurses could accept the method of using the tool or not, notwithstanding the evaluation completion time. Since this study was only conducted in a short period of time, nurses may not fully understand and appreciate that the 3D-DST can standardize the assessment process and improve the recognition rate and accuracy of the delirium assessment. In the future, the duration that the nurses use the 3D-DST should be extended before the acceptance evaluation. Furthermore, the intention to use the 3D-DST and satisfaction perceived by nurses may be affected by organizational factors.

Our study found that the 3D-DST had a slightly higher successful completion rate than the 3D-CAM paper version in assessing delirium among bedside nurses (212/216, 98.1% vs 203/216, 94%; *P*=.045). In the 3D-DST, reminders of the correct responses for items 1, 2 and 5 (objectively testing patients’ cognition) were incorporated into the app, which can increase the accuracy of the delirium assessment, reduce the information processing time, and improve nurses’ work efficiency. When nurses evaluate delirium using the 3D-DST, the system will automatically record and generate the evaluation results without manual input; this prevented possible human errors that are introduced by the nurses. The 3D-DST also added prompts that aimed to reduce the assessment failure rate caused by incorrect or inadequate understanding of the content of the items. By integrating prompts into the 3D-DST, it may have contributed to the reduced failure rate among bedside nurses by guiding and standardizing the assessment process; thus, insufficient knowledge and possible human errors are fully addressed during the assessment. Therefore, the 3D-DST improved the success rate of the delirium assessment. With assistance of CDSSs, decision-making can be incorporated into the routine assessment to guide nurses to complete the assessment successfully with standardized procedures [33-35].

The advantages of the study are multifactorial. The 3D-DST was developed to solve the problems associated with the use of 3D-CAM paper version in routine practice by nurses. The research team included multidisciplinary members such as delirium assessment experts and software development engineers, and we used a combination of multiple architectures to ensure the stability of the system during the development process. In addition, in evaluating the usability and performance (completion rate, time required for completing the assessment, and the number of human errors made by nurses during the assessment) of the 3D-DST, both subjective and objective approaches were used by comparing the 3D-CAM paper version with the 3D-DST among bedside nurses, which showed the promising results of the 3D-DST.

This study also has several limitations. First, each bedside nurse only used the 3D-CAM to evaluate 6 patients, which may have limited the nurses’ possibility to evaluate all types of patients with different admission diagnoses in general wards. Therefore, our result may not be generalizable to other clinical settings. Second, this study only applied a quantitative approach to evaluate the usability of the 3D-DST; interviews can be added to explore the usability of the 3D-DST among bedside nurses in the future. Third, during the usability testing phase, some usability issues in our system may not be adequately reflected due to limitations in the patients’ admission types and the limited number of assessments. Fourth, bedside nurses were not physically involved in the interface design process, which may have affected the usability of the interface. However, we have examined the problems and issues associated with the use of the 3D-CAM paper version among bedside nurses, and the 3D-DST was developed to address these problems by including experienced nursing researchers during the development process, so the results should not be affected. Finally, this study did not analyze the accuracy of the 3D-DST in assessing delirium, as this was not required for the study objectives at this stage, and it was completed as a separate study.

### Conclusion

This study demonstrated that the 3D-DST was perceived as highly acceptable and useful in assisting bedside nurses to identify delirium among older adults in routine practice. The integration of this app with existing health systems could enhance its positive impact on the efficiency and accuracy of delirium screening in the future.

## Supplementary material

10.2196/51264 Multimedia Appendix 1 Details of the design principle.

10.2196/51264 Multimedia Appendix 2 Evaluation process of the original 3-Minute Diagnostic Interview for Confusion Assessment Method–Defined Delirium (3D-CAM) paper version.

10.2196/51264 Multimedia Appendix 3 Evaluation process of the Delirium Assessment Tool With Decision Support Function Based on the 3-Minute Diagnostic Interview for Confusion Assessment Method–Defined Delirium (3D-DST).

10.2196/51264 Checklist 1 CONSORT-eHEALTH checklist (V 1.6.1).

## Abbreviations

3D-CAM: 3-Minute Diagnostic Interview for Confusion Assessment Method–Defined Delirium
3D-DST: Delirium Assessment Tool With Decision Support Function Based on the 3-Minute Diagnostic Interview for Confusion Assessment Method–Defined Delirium
CAM: Confusion Assessment Method
CAM-ICU: Confusion Assessment Method for Intensive Care Unit
CDSS: clinical decision support system
ICU: intensive care unit
MVVM: model-view-viewmodel
NIS: nursing information system
