# Supplementary material for: Development and Usability Testing of a Mobile App–Based Clinical Decision Support System for Delirium: Randomized Crossover Trial
Source: JMIR Aging. 2024 Jan 24;7:e51264. doi: 10.2196/51264 (PMC10850851; doi:10.2196/51264)
Supplement: Multimedia Appendix 1 [file aging-v7-e51264-s001.docx]

Details of the design principle

The overall goal of the 3D-DST was to design an automated delirium assessment system that was suitable for general ward nurses. It was expected to be reliable and scalable, with safe performance, a friendly interface, and convenient operation, also was fast and accurate in completing the delirium assessment.

1. Simple, natural, and consistent: the design of user interfaces should avoid visual clutter, display concise information, include necessary functions to complete the assessment. In addition, the system should be familiar and easy to use for bedside nurses. Consistency included internal consistency and external consistency, internal consistency meant that the system always used a consistent concept, process, appearance, and layout. External consistency referred to the interaction behavior and experience of nurses when they used the system, which needed to remain as consistent as possible with the interaction behavior and experience of using other software or applications.
2. Use concise terms: all terms used in the system should be concise and to the point, and they are familiar, meaningful and relevant to nurses’ routine work. Abbreviations and acronyms should be clear and only be used when they are easy to understand.
3. Minimization of cognitive load: when designing the system interfaces, the content should be presented in the most direct way, avoiding excessive information transmission and display, as well as insufficient information prompt.
4. Fault tolerance and providing valuable feedback: this referred to that the system was designed to allow end-users to make mistakes but was able to remind the end-users and provide feedback timely. Providing valuable feedback to end-users required clear navigation to let nurses know what assessments they were currently conducting and what they would be doing, this ensured that nurses can use the system to complete the assessments successfully. The system allowed nurses to modify the assessment data if necessary, or return to the previous assessment during the process. Fault tolerance and feedback work together to reduce human errors and provide valuable feedback before errors occur.
5. Appropriate information display: when designing the interface of the system, besides providing all necessary information, appropriate interface information display should be maintained by limiting too much information to be displayed or avoiding change too fast. Due to the fast-paced working environment and workload among nurses, it is necessary to only display the most critical information and maintain reasonable layout in the information interfaces.
6. Storage and input of information: the system allowed continuously and automatically store, input data and retrieve necessary information, so nurses can directly view the stored information at any time, such as viewing the history of patient assessments.
7. Efficient interaction design: this allowed nurses to use the system completing the assessment successfully, with the shortest, simplest, and the most direct operations.
8. Intelligentization: this referred to the use of intellectual functions such as automatic calculation, judgment, recognition, memorization, reminder, rapid extraction of relevant information, rapid analysis, and auxiliary decision-making within the information system to assist delirium assessment.
