# Supplementary figures and images for: Development and Usability Testing of a Mobile App–Based Clinical Decision Support System for Delirium: Randomized Crossover Trial
Source: JMIR Aging. 2024 Jan 24;7:e51264. doi: 10.2196/51264 (PMC10850851; doi:10.2196/51264)

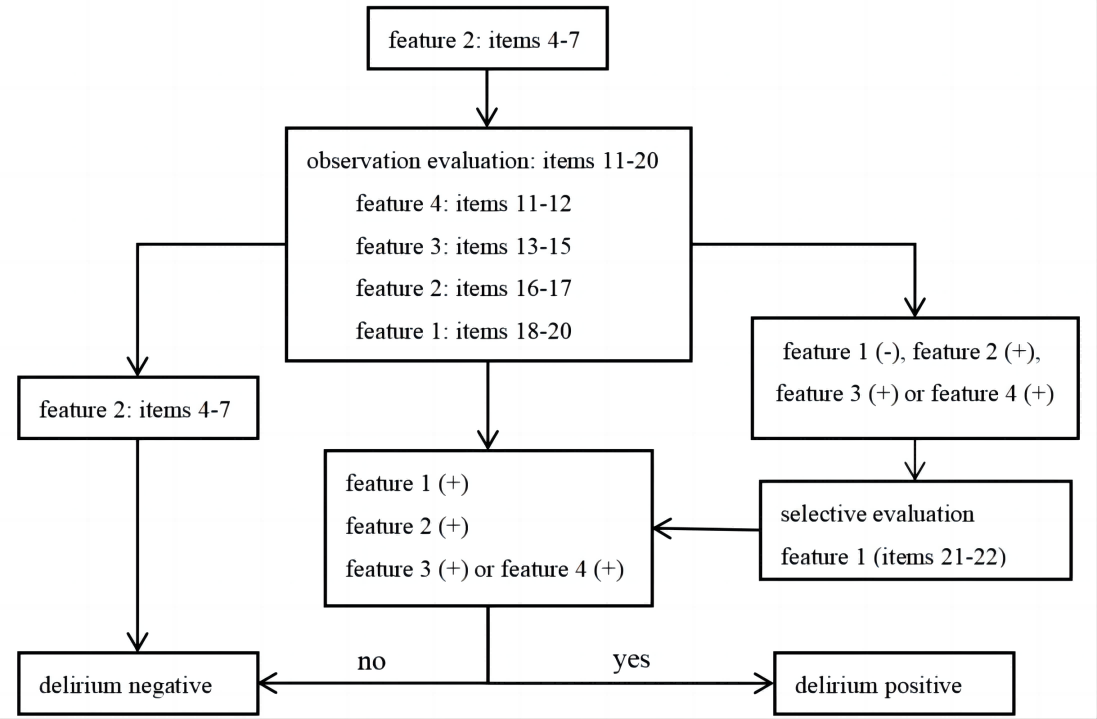

Supplement: Multimedia Appendix 2 [file aging-v7-e51264-s002.png]

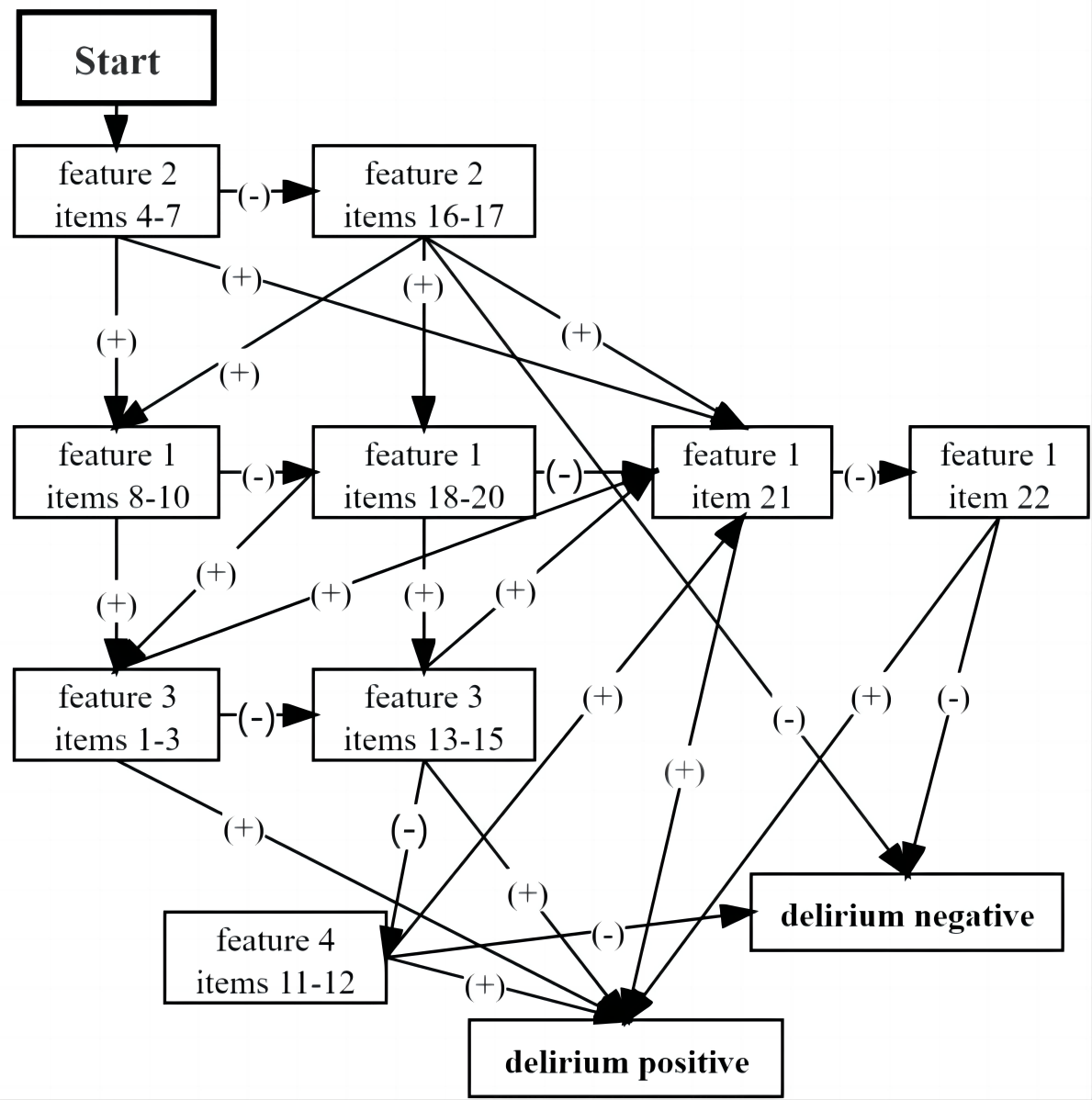

Supplement: Multimedia Appendix 3 [file aging-v7-e51264-s003.png]
